# Kinetic prediction of reverse intersystem crossing in organic donor–acceptor molecules

**DOI:** 10.1038/s41467-020-17777-2

**Published:** 2020-08-06

**Authors:** Naoya Aizawa, Yu Harabuchi, Satoshi Maeda, Yong-Jin Pu

**Affiliations:** 1 RIKEN Center for Emergent Matter Science (CEMS), 2-1 Hirosawa, Wako, Saitama 351-0198 Japan; 2 Precursory Research for Embryonic Science and Technology (PRESTO), Japan Science and Technology Agency (JST), 4-1-8 Honcho, Kawaguchi, Saitama 332-0012 Japan; 3 Department of Chemistry, Faculty of Science, Hokkaido University, Kita 10, Nishi 8, Kita-ku, Sapporo 060-0810 Japan; 4 Institute for Chemical Reaction Design and Discovery (WPI-ICReDD), Hokkaido University, Kita 21 Nishi 10, Kita-ku, Sapporo, Hokkaido 001-0021 Japan

**Keywords:** Photochemistry, Organic LEDs

## Abstract

Reverse intersystem crossing (RISC), the uphill spin-flip process from a triplet to a singlet excited state, plays a key role in a wide range of photochemical applications. Understanding and predicting the kinetics of such processes in vastly different molecular structures would facilitate the rational material design. Here, we demonstrate a theoretical expression that successfully reproduces experimental RISC rate constants ranging over five orders of magnitude in twenty different molecules. We show that the spin flip occurs across the singlet–triplet crossing seam involving a higher-lying triplet excited state where the semi-classical Marcus parabola is no longer valid. The present model explains the counterintuitive substitution effects of bromine on the RISC rate constants of previously unknown molecules, providing a predictive tool for material design.

## Abstract (web summary)

Understanding and predicting the kinetics of reverse intersystem crossing (RISC) facilitates the design of materials. Here, the authors demonstrate a theoretical expression that reproduces experimental RISC rate constants ranging over five orders of magnitude in selected molecules.

## Introduction

Electronic spin-flip processes in molecular excited states have attracted increasing interest for optoelectronics^1–3^, photocatalytic synthesis^4–6^, and biomedical applications^7–9^. A relevant example is reverse intersystem crossing (RISC), the uphill transition of a non-emissive triplet excited state to an emissive singlet excited state. This process leads to E-type delayed fluorescence, also known as thermally activated delayed fluorescence (TADF), and allows an internal charge-to-photon conversion efficiency of nearly 100% in organic light-emitting diodes^10^. Although materials have typically been discovered experimentally, a fundamental understanding of RISC kinetics and strategy for predicting the rate constants may open vast opportunities for theory-driven materials discovery.

**Fig. 1:**
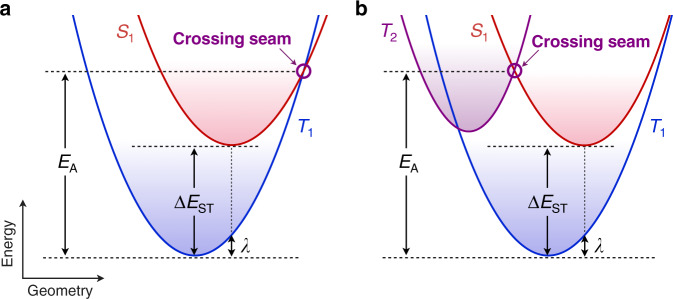
RISC from triplet to singlet excited states. **a**, **b** Schematic potential energy surfaces of excited states depicting RISC via (**a**) a S_1_–T_1_ crossing seam and (**b**) a S_1_–T_2_ crossing seam.

RISC kinetics are often considered in the framework of Marcus theory^11–14^. If the spin–orbit coupling *H*_SO_ between the initial triplet and final singlet excited states is weak, meaning that the spin-flip only occurs on the crossing seam between their potential energy surfaces (PESs) (Fig. 1a), the RISC rate constant (*k*_RISC_) follows a Marcus-like nonadiabatic expression:

1 $$k_{{\mathrm{RISC}}} = \frac{{2\pi }}{\hbar }\left| {H_{{\mathrm{SO}}}^{}} \right|^2\left( {4\pi \lambda k_{\mathrm{B}}T} \right)^{ - \frac{1}{2}}{\mathrm{exp}}\left( {\frac{{ - E_{\mathrm{A}}}}{{k_{\mathrm{B}}T}}} \right)$$

where *ћ* is the reduced Planck constant, *k*_B_ is the Boltzmann constant, *T* is the temperature, *λ* is the reorganization energy, and *E*_A_ is the activation energy to reach the crossing seam. In the case of simple parabolic PESs with equal force constants, which is a crucial assumption of Marcus theory, *E*_A_ can be analytically expressed as

2 $$E_A = \frac{{\left( {\Delta E_{{\mathrm{ST}}} + \lambda } \right)^2}}{{4\lambda }}$$

with Δ*E*_ST_ as the adiabatic singlet–triplet energy difference. A key implication of Eqs. (1) and (2) is that *k*_RISC_ can be predicted from the equilibrium geometries, which correspond to the easily computable local minima on the PESs of the initial triplet state and final singlet state. However, this understanding of RISC becomes more complicated if the spin-flip process involves an energetically higher-lying excited state as an intermediate^15–19^ (Fig. 1b). Since Eq. (2) does not include information on the key intermediate involved in the actual spin-flip process, recent calculations using the equilibrium geometries only provided qualitative justification of the experimental *k*_RISC_ for a handful of TADF molecules^20^. Herein, we explicitly compute singlet–triplet crossing seams to quantitatively predict *k*_RISC_ for vastly different structures both from the literature and previously unknown molecules. Rigorous comparisons to experimental data reported over the last decade allowed a general understanding of the RISC kinetics governed by the singlet–triplet crossing seam involving a higher-lying triplet excited state.

## Results

### Computation of *k*_RISC_

**Fig. 2:**
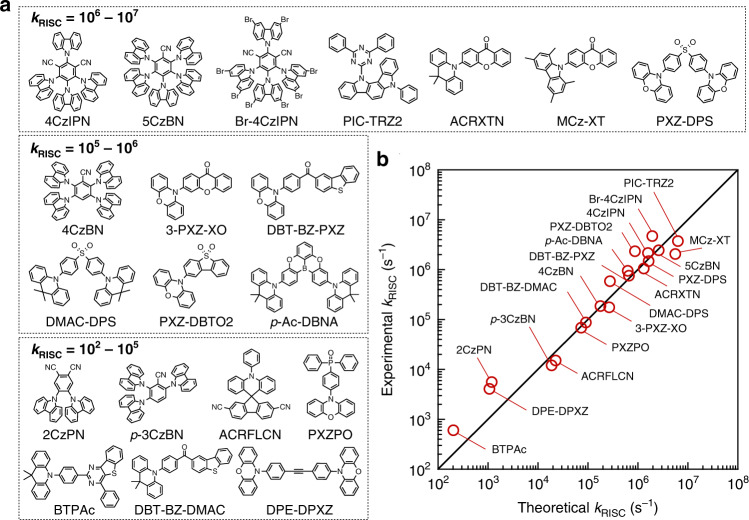
Twenty molecules examined in this study and their *k*_RISC_ values. **a** Molecular structures of the examined TADF materials categorized by their *k*_RISC_ values. **b** Comparison of the experimental and theoretical *k*_RISC_ values.

To understand and predict the RISC kinetics, we first focused on twenty different TADF molecules reported in the literature (Fig. 2a). These molecules are characterized as donor–acceptor systems in which electron-rich donor units, aryl amines, are covalently bound to electron-deficient acceptor units, such as heterocycles, aryl nitriles, ketones, boranes, sulfones, alkynes, or phosphine oxides. We collected literature values for the steady-state and transient photoluminescence data of these molecules and estimated their *k*_RISC_ values from differential rate equations of the population densities of their singlet and triplet excited states (see Supplementary Information Section 1 for details). The experimental *k*_RISC_ values varied substantially, by five orders of magnitude, from 10^2^ to 10^7^ s^–1^.

Directly computing *k*_RISC_ from Eq. (1) requires the minimum-energy seam of the crossing (MESX), the energetically most accessible geometry on the singlet–triplet crossing seam hypersurface^21,22^, as well as equilibrium excited-state geometries. To obtain the MESX for each molecule, we employed a constrained optimization algorithm using the gradient projection method^23^, which minimizes the mean energy of the singlet and triplet states (*E*_S_ + *E*_T_)/2 while simultaneously fulfilling the crossing condition of the energy difference *E*_S_ − *E*_T_ = 0. *E*_S_ and *E*_T_ were calculated at the level of time-dependent density functional theory (TDDFT) within the Tamm–Dancoff approximation^24^ (see the Methods for details).

For the MESX geometries of the twenty molecules shown in Fig. 2a, TDDFT predicts nonzero *H*_SO_ of 0.17–3.61 cm^–1^ with fairly small *E*_A_ of 0.11–0.32 eV, corresponding to *k*_RISC_ of 10^2^–10^7^ s^–1^ calculated using Eq. (1) at *T* of 300 K. Figure 2b compares the theoretical *k*_RISC_ values to the experimental values, demonstrating that the present model successfully reproduces the experimental rates. The mean absolute logarithmic error (MALE) reaches only 0.23, whereas a larger MALE of 1.2, corresponding to an error of 1.2 orders of magnitude, is observed for the values based on the conventional model shown in Fig. 1a and the parabolic approximation of Eq. (2) (see Supplementary Fig. 1 for the errors for each molecule). These results thus demonstrate the importance of the explicit computation of the singlet–triplet crossing seams for quantitatively predicting *k*_RISC_.

### Mechanism of the RISC

Closer inspection of the data further reveals that the lowest singlet excited state (S_1_) does not cross the lowest triplet state (T_1_) and instead crosses the higher-lying triplet state (T_2_) at the obtained MESX geometry, consistent with the model shown in Fig. 1b. This feature explains the larger errors for the parabolic approximation, which does not account for any higher-lying excited states. We attribute the uncrossed S_1_ and T_1_ to a nonzero exchange interaction between the singlet and triplet states, which leads to T_1_ always lying below S_1_ if the two states have the same electronic configuration^25^. In accordance with El-Sayed’s rule^26^, a large change in the orbital angular momentum between S_1_ and T_2_ consisting of different electronic configurations induces an effective *H*_SO_ and thus enables spin flipping via the MESX. These results are consistent with the RISC picture anticipated based on recent theoretical and experimental studies using ACRXTN^16^ and 4CzIPN^19^. It must be stressed that S_1_–T_2_ MESX is present in every molecule examined in this quantitative study despite their wide variety of excited-state electronic configurations, including intramolecular charge transfer (CT) states and locally excited (LE) states of π–π^*^ and *n*–π^*^ on either donor or acceptor units, illustrating the generality of RISC via S_1_–T_2_ crossing in organic donor–acceptor molecules.

### Prediction of the effects of bromination on *k*_RISC_

**Fig. 3:**
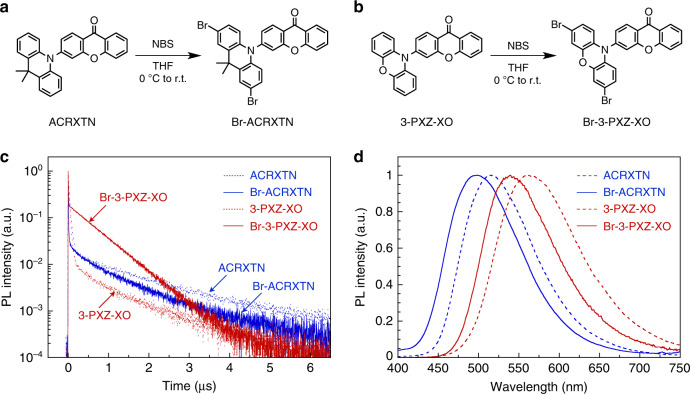
Synthesis and photoluminescence properties of the brominated molecules. **a**, **b** Synthetic routes to Br-ACRXTN (**a**) and Br-3-PXZ-XO (**b**). **c**, **d** Transient photoluminescence decays (**c**) and steady-state photoluminescence spectra (**d**) of ACRXTN, Br-ACRXTN, 3-PXZ-XO, and Br-3-PXZ-XO in a solid-state host matrix, 2,8-bis(diphenylphosphoryl)dibenzo[*b*,*d*]furan (PPF), at a doping concentration of 5 wt%.

To further validate the present RISC model of Eq. (1), we computed *k*_RISC_ of brominated analogues of representative TADF materials ACRXTN and 3-PXZ-XO: 3-(2,7-dibromo-9,9-dimethylacridan-10-yl)xanthone (Br-ACRXTN) and 3-(3,7-dibromo-phenoxazin-10-yl)xanthone (Br-3-PXZ-XO) (Fig. 3a, b). Although heavy halogen atoms such as bromine are well known to induce large *H*_SO_ and thus facilitate ISC^27^, the calculations predict that the electrophilic bromination of ACRXTN counterintuitively decreases *k*_RISC_ from 1.3 × 10^6^ s^–1^ to 7.1 × 10^5^ s^–1^. In contrast, the bromination of 3-PXZ-XO leads to a more than hundredfold increase in *k*_RISC_ from 2.7 × 10^5^ s^–1^ to 4.2 × 10^7^ s^–1^. Indeed, subsequent synthesis and characterization confirm the predicted opposite trend; the brominations of ACRXTN and 3-PXZ-XO caused the experimental *k*_RISC_ to decrease from 1.0 × 10^6^ s^–1^ to 8.7 × 10^5^ s^–1^ and to increase from 1.7 × 10^5^ s^–1^ to 2.6 × 10^7^ s^–1^, respectively (see Supplementary Fig. 2 and Table S1 for details). To the best of our knowledge, *k*_RISC_ of over 10^7^ s^–1^ for Br-3-PXZ-XO is the highest value ever reported for an organic TADF material^28^. This high *k*_RISC_ reflects its fast transient photoluminescence decay with a delayed fluorescence lifetime of 490 ns (Fig. 3c), which is considerably shorter than typical values of several microseconds^29^. We also note that both brominated molecules exhibit similar blueshifts in their broad, unstructured CT emissions compared to the corresponding nonbrominated analogues (Fig. 3d), and this shift is attributed to the electron-withdrawing effects of the bromine atoms on the donor units, destabilizing the CT states between the donor and acceptor units (i.e., increasing the energy of the CT states).

**Fig. 4:**
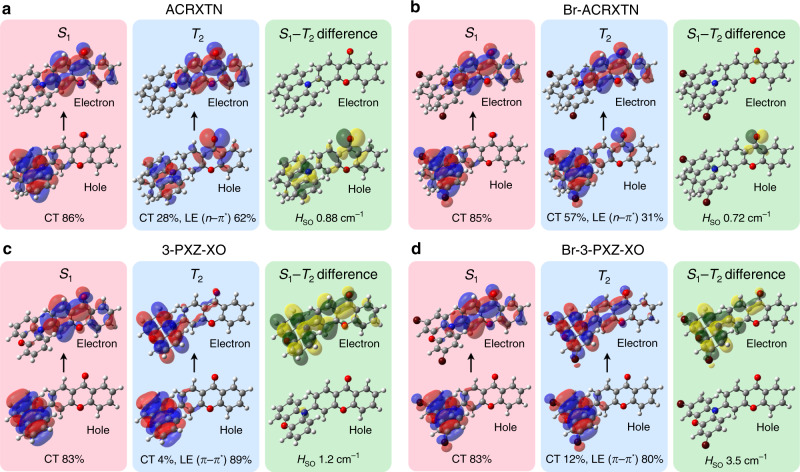
Electronic configurations of S_1_–T_2_ MESXs. **a–d** Natural transition orbitals (NTOs) for the excited states of ACRXTN (**a**), Br-ACRXTN (**b**), 3-PXZ-XO (**c**), and Br-3-PXZ-XO (**d**) at S_1_–T_2_ MESX geometries. The differences in the density of the S_1_ and T_2_ NTOs are also shown.

The notable retardation of *k*_RISC_ by bromination of ACRXTN is due to a decrease in *H*_SO_ from 0.88 cm^–1^ to 0.72 cm^–1^ at the S_1_–T_2_ MESX geometries. This counterintuitive substitution effect of bromine on *H*_SO_ can be rationalized by two factors. First, the S_1_–T_2_ spin flipping in Br-ACRXTN is compensated by a smaller change in the orbital angular momentum than that in ACRXTN (Fig. 4a, b). This is due to an increase in the occupation of the CT state in T_2_ from 28% to 57% upon bromination, which leads to both S_1_ and T_2_ having similar CT states with small *H*_SO_ according to El-Sayed’s rule. Additionally, the resulting change in the orbital angular momentum of Br-ACRXTN involves the *n* orbital of the carbonyl oxygen on the acceptor unit rather than bromine on the donor unit (Fig. 4b), suggesting that the heavy atom effect plays a minor role in determining *H*_SO_ between S_1_ and T_2_. In contrast, Br-3-PXZ-XO has a perceivable contribution from the bromine atom to the orbital angular momentum change between S_1_ of the CT state and T_2_ of the LE π–π^*^ state on the donor unit (Fig. 4c, d). Such circumstances are indeed consistent with the heavy atom effect of bromine being responsible for the increase in *H*_SO_ from 1.2 cm^–1^ to 3.5 cm^–1^ and thus for the high *k*_RISC_, over 10^7^ s^–1^, in Br-3-PXZ-XO.

## Discussion

**Fig. 5:**
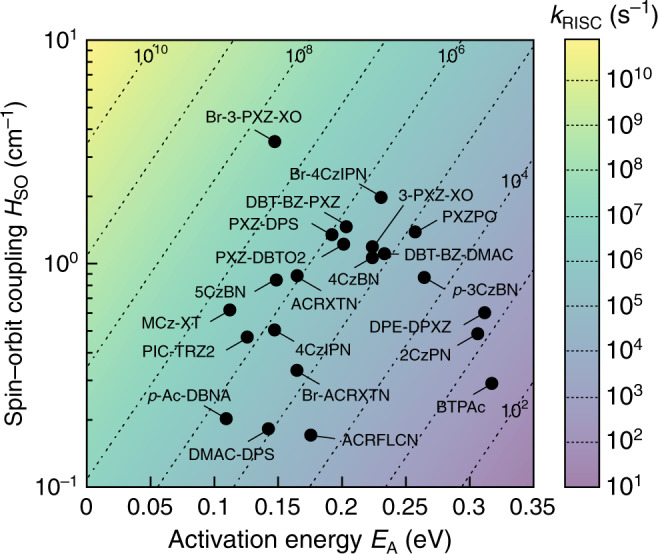
Overview of theoretical *k*_RISC_. *k*_RISC_ as a function of *H*_SO_ and *E*_A_ with a fixed *λ* of 0.10 eV and *T* of 300 K. The *H*_SO_ and *E*_A_ values of the examined molecules are also plotted.

Figure 5 displays the impact of varying *H*_SO_ and *E*_A_ on *k*_RISC_. While the existing organic TADF molecules exhibit *k*_RISC_ smaller than 10^8^ s^–1^, the theory predicts that even *k*_RISC_ of 10^9^ s^–1^, corresponding to a time constant of 1.0 ns, can be achieved with *H*_SO_ less than 10 cm^–1^; for example, *H*_SO_ of 7.7 cm^–1^ for *E*_A_ of 0.10 eV and *H*_SO_ of 2.9 cm^–1^ for *E*_A_ of 0.05 eV at *T* of 300 K. These *H*_SO_ are an order of magnitude smaller than those of iridium-containing phosphors and could be achieved by exploiting heavy atom effects of nonmetals in periods 3 and 4^30,31^. However, we have shown that such heuristic approaches sometimes lead to the retardation of *H*_SO_, in part because of their more pronounced effects on the excited-state electronic configurations at the S_1_–T_2_ MESX geometries. Thus, for material design, a priori computational screening is essential, and the RISC model presented here allows for it.

In summary, we have presented a RISC kinetic model that successfully predicts the experimental rates for a wide variety of organic TADF molecules. Our results suggest that explicitly computing the crossing seam between the singlet and triplet excited states leads to more reliable predictions than those obtained by the conventional approach using the Marcus parabolic approximation because the RISC in these molecules involves higher-lying triplet excited states. The presented model is thus a viable tool for theory-driven materials discovery with a relevant exemplar exhibiting a high *k*_RISC_ of 2.6 × 10^7^ s^–1^.

We envisage that further computational screenings of vast chemical space will facilitate the discovery of materials exploiting the spin-flipping process for various photochemical applications. We also anticipate the possible existence of materials that may not follow the model and provide platforms to discover spin-flipping mechanisms different from the presented one.

## Methods

### Computation

The geometries of the singlet–triplet MESXs, where the square energy difference (*E*_S_ − *E*_T_)^2^ and the mean energy (*E*_S_ + *E*_T_)/2 are minimized, were obtained by the gradient projection method^23^ using a composed gradient vector **G** for the nuclear coordinates **Q**:

3 $${\mathbf{G}}\left( {\mathbf{Q}} \right) = 2\left( {E_{\mathrm{S}}\left( {\mathbf{Q}} \right)-E_{\mathrm{T}}\left( {\mathbf{Q}} \right)} \right)\frac{{\mathbf{v}}}{{\left| {\mathbf{v}} \right|}} + \frac{1}{2}\left( {\frac{{\partial E_{\mathrm{S}}\left( {\mathbf{Q}} \right)}}{{\partial {\mathbf{Q}}}} + \frac{{\partial E_{\mathrm{T}}\left( {\mathbf{Q}} \right)}}{{\partial {\mathbf{Q}}}}} \right){\mathbf{P}}$$

where

4 $${\mathbf{P}} = 1 - \frac{{{\mathbf{vv}}^{\mathrm{T}}}}{{\left| {\mathbf{v}} \right|^2}}$$

In Eq. (3), the first term contains the difference gradient vector **v** to minimize the energy difference. The second term is responsible for minimizing the mean energy, while the projection matrix **P** ensures the orthogonality between the two terms of the composed gradient vector. The excited-state energy and gradient were calculated using linear-response TDDFT with the LC-BLYP functional^32^ and the 6–31+G(d) basis set within the Tamm–Dancoff approximation^24^. The range-separated parameters for the LC-BLYP functional were non-empirically optimized for each molecule to incorporate a reasonable amount of exact exchange^33,34^. The geometry optimization of the MESXs was performed with the GRRM17 program^35^, which refers to the energy and gradient calculated by the Gaussian 16 program^36^. The *H*_SO_ values were calculated perturbatively using the Breit–Pauli spin–orbit Hamiltonian with an effective charge approximation implemented in the PySOC program^37^ interfaced to the Gaussian 16 program. The *E*_A_ values were calculated as the electronic energy difference between the MESX and the equilibrium T_1_. The *λ* values were calculated as the difference between the T_1_ electronic energies at the equilibrium T_1_ and S_1_ geometries.

### Chemical synthesis

The synthetic procedures and characterization data of the compounds are detailed in Supplementary Information Section 1.

### Photoluminescence measurements

Steady-state photoluminescence spectra were acquired using a Fluoromax-4 spectrophotometer (HORIBA) with 370 nm photoexcitation from a Xe arc lamp. Transient photoluminescence decay measurements were performed by time-correlated single photon counting under a flow of N_2_ using a Fluorolog-3 fluorescence lifetime spectrometer (HORIBA) with a 370 nm LED excitation source. The absolute PL quantum yields were determined under a flow of N_2_ using a C9920 integrating sphere system (Hamamatsu Photonics). The method for determining the experimental *k*_RISC_ values is detailed in Supplementary Information Section 1.

## Supplementary information

Supplementary Information

Peer Review File

## Data Availability

The data that support the findings of this study available in this published Article and its Supplementary Information, or from the corresponding authors upon reasonable request.
