# Supplementary material for: Kinetic prediction of reverse intersystem crossing in organic donor–acceptor molecules
Source: Nat Commun. 2020 Aug 6;11:3909. doi: 10.1038/s41467-020-17777-2 (PMC7411052; doi:10.1038/s41467-020-17777-2)
Supplement: Supplementary file 1 — Supplementary Information [file 41467_2020_17777_MOESM1_ESM.pdf]

Supplementary Information

Kinetic Prediction of Reverse Intersystem Crossing in Organic Donor–Acceptor Molecules

Naoya Aizawa,[1,2]* Yu Harabuchi,[2,3,4]* Satoshi Maeda[3,4] and Yong-Jin Pu[1]

[1] RIKEN Center for Emergent Matter Science (CEMS), 2-1 Hirosawa, Wako, Saitama, 351-0198, Japan.

[2] Precursory Research for Embryonic Science and Technology (PRESTO), Japan Science and Technology Agency (JST), 4-1-8 Honcho, Kawaguchi, Saitama 332-0012, Japan.

[3] Department of Chemistry, Faculty of Science, Hokkaido University, Kita 10, Nishi 8, Kita-ku, Sapporo 060-0810, Japan.

[4] Institute for Chemical Reaction Design and Discovery (WPI-ICReDD), Hokkaido University, Kita 21 Nishi 10, Kita-ku, Sapporo, Hokkaido 001-0021, Japan.

Corresponding Authors: E-mail: naoya.aizawa@riken.jp (N.A.); y_harabuchi@sci.hokudai.ac.jp (Y.H.)

Table of contents

1. Supplementary Method

Supplementary Table 1 Photoluminescence properties of the examined TADF materials

Supplementary Figure 1 Comparison of the logarithmic errors between the experimental and theoretical k_{RISC} values based on the minimum-energy seam of crossing (MESX) and the parabolic approximation

Supplementary Figure 2 Comparison of the experimental and theoretical k_{RISC} values

Supplementary Figure 3 ^{1}H NMR spectrum of Br-ACRXTN (400 MHz, $CDCl_3$, 293 K)

Supplementary Figure 4 Magnified view of the ^{1}H NMR spectrum of Br-ACRXTN (400 MHz, $CDCl_3$, 293 K)

Supplementary Figure 5 ^{13}C NMR spectrum of Br-ACRXTN (100 MHz, $CDCl_3$, 293 K)

Supplementary Figure 6 ^{1}H NMR spectrum of Br-3-PXZ-XO (400 MHz, $CDCl_3$, 293 K)

Supplementary Figure 7 Magnified view of the ^{1}H NMR spectrum of Br-3-PXZ-XO (400 MHz, $CDCl_3$, 293 K)

Supplementary Figure 8 ^{13}C NMR spectrum of Br-3-PXZ-XO (100 MHz, $CDCl_3$, 293 K)

2. Supplementary References

1. Supplementary Method

Determination of experimental rate constants of reverse intersystem crossing (k_{RISC}). The rate equation for the densities of singlet and triplet excited states, $[\mathrm{S_1}]$ and $[\mathrm{T_1}]$, is expressed as

$$\frac{\mathrm{d}}{\mathrm{d}t}\begin{pmatrix}[\mathrm{S_1}]\\ [\mathrm{T_1}]\end{pmatrix}=\begin{pmatrix}-(k_{\mathrm{r,S}}+k_{\mathrm{nr,S}}+k_{\mathrm{ISC}}) & k_{\mathrm{RISC}}\\ k_{\mathrm{ISC}} & -(k_{\mathrm{r,T}}+k_{\mathrm{nr,T}}+k_{\mathrm{RISC}})\end{pmatrix}\begin{pmatrix}[\mathrm{S_1}]\\ [\mathrm{T_1}]\end{pmatrix} \quad (1)$$

where $k_{\mathrm{r,S}}$, $k_{\mathrm{nr,S}}$ and k_{ISC} are the rate constants of radiative decay (fluorescence), nonradiative decay to the ground state ($\mathrm{S_0}$) and intersystem crossing (ISC) of the singlet excited state ($\mathrm{S_1}$), respectively. $k_{\mathrm{r,T}}$, $k_{\mathrm{nr,T}}$ and k_{RISC} are the rate constants of radiative decay (phosphorescence), nonradiative decay to $\mathrm{S_0}$ and reverse ISC (RISC) of the triplet excited state ($\mathrm{T_1}$), respectively. The solution to Equation 1 is a biexponential decay given by

$$[\mathrm{S_1}]=A_1\exp\left[-k_{\mathrm{PF}}t\right]+A_2\exp\left[-k_{\mathrm{DF}}t\right] \quad (2)$$

where A_1 and A_2 are the amplitudes (exponential prefactors). The measurable rate constants of prompt fluorescence and delayed fluorescence (k_{PF} and k_{DF}) are thus given by

$$k_{\mathrm{PF}},k_{\mathrm{DF}}=\frac{1}{2}\left(k_{\mathrm{r,S}}+k_{\mathrm{nr,S}}+k_{\mathrm{ISC}}+k_{\mathrm{r,T}}+k_{\mathrm{nr,T}}+k_{\mathrm{RISC}}\right)\times\left(1\pm\sqrt{1-\frac{4\left(k_{\mathrm{r,S}}+k_{\mathrm{nr,S}}+k_{\mathrm{ISC}}\right)\left(k_{\mathrm{r,T}}+k_{\mathrm{nr,T}}+k_{\mathrm{RISC}}\right)-4k_{\mathrm{ISC}}k_{\mathrm{RISC}}}{\left(k_{\mathrm{r,S}}+k_{\mathrm{nr,S}}+k_{\mathrm{ISC}}+k_{\mathrm{r,T}}+k_{\mathrm{nr,T}}+k_{\mathrm{RISC}}\right)^2}}\right) \quad (3)$$

The measurable PL quantum yields of prompt fluorescence and delayed fluorescence (Φ_{PF} are Φ_{DF}) are expressed as

$$\Phi_{\mathrm{PF}}=\frac{k_{\mathrm{r,S}}}{k_{\mathrm{r,S}}+k_{\mathrm{nr,S}}+k_{\mathrm{ISC}}} \quad (4)$$

$$\Phi_{\mathrm{DF}}=\sum_{n=1}^{\infty}\Phi_{\mathrm{PF}}(\Phi_{\mathrm{ISC}}\Phi_{\mathrm{RISC}})^n=\frac{\Phi_{\mathrm{PF}}\Phi_{\mathrm{ISC}}}{1-\Phi_{\mathrm{ISC}}} \quad (5)$$

Assuming (i) $k_{\mathrm{r,S}}$, $k_{\mathrm{ISC}} >> k_{\mathrm{nr,S}}$, k_{RISC} and (ii) $k_{\mathrm{RISC}} >> k_{\mathrm{r,T}}$, $k_{\mathrm{nr,T}}$, Equations 3, 4, and 5 yield

$$k_{\mathrm{r,S}}=k_{\mathrm{PF}}\Phi_{\mathrm{PF}} \quad (6)$$

$$k_{\mathrm{ISC}}=k_{\mathrm{PF}}-k_{\mathrm{r,S}}=k_{\mathrm{PF}}\left(1-\Phi_{\mathrm{PF}}\right) \quad (7)$$

$$k_{\mathrm{RISC}}=\frac{k_{\mathrm{PF}}k_{\mathrm{DF}}\Phi_{\mathrm{DF}}}{k_{\mathrm{ISC}}\Phi_{\mathrm{PF}}}=\frac{k_{\mathrm{DF}}\Phi_{\mathrm{DF}}}{\Phi_{\mathrm{PF}}\left(1-\Phi_{\mathrm{PF}}\right)} \quad (8)$$

We estimated experimental k_{RISC} values using Equation 8 from the literature values of k_{PF}, k_{DF}, Φ_{PF}, and Φ_{DF} for 2,4,5,6-tetra(carbazol-9-yl)isophthalonitrile (4CzIPN)[1], 2,3,5,6-tetra(carbazol-9-yl)benzonitrile (4CzBN)[1], 2,3,6-tri(carbazol-9-yl)benzonitrile (*p*-3CzBN)[1], 2,3,4,5,6-penta(carbazol-9-yl)benzonitrile (5CzBN)[2], 4,5-di(carbazol-9-yl)phthalonitrile (2CzPN)[3], 12-(4,6-diphenyl-1,3,5-triazin-2-yl)-5-phenyl-5,12-dihydroindolo[3,2-*a*]carbazole (PIC-TRZ2)[4], 3-(1,3,6,8-tetramethylcarbazol-9-yl)xanthone (MCz-XT)[5], bis(4-(phenoxazin-10-yl)phenyl)sulfone (PXZ-DPS)[6], bis(4-(9,9-dimethylacridan-10-yl)phenyl)sulfone (DMAC-DPS)[7], dibenzo[*b,d*]thiophen-2-yl(4-(phenoxazin-10-yl)phenyl)methanone (DBT-BZ-PXZ)[8], dibenzo[*b,d*]thiophen-2-yl(4-(9,9-dimethylacridan-10-yl)phenyl)methanone (DBT-BZ-DMAC)[8], 2-(phenoxazin-10-yl)dibenzo[*b,d*]thiophene-5,5-dioxide (PXZ-DBTO2)[9], 3,11-bis(9,9-dimethylacridan-10-yl)-5,9-dioxa-13b-boranaphtho[3,2,1-*de*]anthracene (*p*-Ac-DBNA)[10], 10-phenyl-10*H*-spiro[acridine-9,9′-fluorene]-2′,7′-dicarbonitrile (ACRFLCN)[11], (4-(phenoxazin-10-yl)phenyl)diphenylphosphine-oxide (PXZXO)[12], 2-(4-(9,9-dimethylacridan-10-yl)phenyl)-4-phenylbenzo[4,5]thieno[3,2-*d*]pyrimidine (BTPAc)[13], and 1,2-bis(4-(phenoxazin-10-yl)phenyl)acetylene (DPE-DPXZ)[14]. The reported value of k_{RISC} was taken directly from the literature for 2,4,5,6-tetra(3,6-dibromocarbazol-9-yl)isophthalonitrile (Br-4CzIPN)[15]. We also performed steady-state and transient photoluminescence measurements on 3-(9,9-dimethylacridan-10-yl)xanthone (ACRXTN)[16], 3-(9,9-dimethylacridan-10-yl)xanthone (3-PXZ-XO)[17], 3-(2,7-dibromo-9,9-dimethylacridan-10-yl)xanthone (Br-ACRXTN), and 3-(3,7-dibromo-phenoxazin-10-yl)xanthone (Br-3-PXZ-XO) in a solid-state host matrix, 2,8-bis(diphenylphosphoryl)dibenzo[*b*,*d*]furan (PPF), at a concentration of 5 wt% and determined their experimental k_{RISC} values, as shown in Table S1. These materials were selected such that the value of k_{RISC} would be diverse. The materials reported to exhibit low $k_{RISC} < 10^5\ s^{-1}$ are limited and were thus chosen with no other specific intent. For high $k_{RISC} > 10^5\ s^{-1}$, we chose representative TADF materials, such as 4CzIPN and ACRXTN, that have been studied by several groups. Additionally, the selection was intended to include various popular acceptor units for TADF materials, such as heterocycles, aryl nitriles, ketones, boranes, sulfones, and phosphine oxides.

Synthesis and characterization. ACRXTN and 3-PXZ-XO were prepared according to the literature procedures[16,17]. ^{1}H and ^{13}C NMR spectra were recorded on a JNM-AL400 (JEOL). Chemical shifts of the ^{1}H

and ^{13}C NMR signals were referenced to the signals of tetramethylsilane and $CDCl_3$, respectively, as internal references. Matrix-assisted laser desorption/ionization time-of-flight (MALDI-TOF) mass spectra were collected on an ULTRAFLEX (Bruker) using dithranol as the matrix. The melting points was measured on a STA7200 (HITACHI). The detailed synthetic procedures for Br-ACRXTN and Br-3-PXZ-XO are described below. All reactions were performed under a N_2 atmosphere.

Synthesis of Br-ACRXTN. To a stirred solution of ACRXTN (0.101 g, 0.250 mmol) in THF (5 mL) was added *N*-bromosuccinimide (0.107 g, 0.600 mmol) at 0 °C. The mixture was stirred at that temperature for 1 hour and slowly warmed to room temperature for 8 hours. The mixture was poured into water and then extracted with CH_2Cl_2. The combined organic layers were concentrated under reduced pressure. The crude product was purified by silica-gel column chromatography (eluent: CH_2Cl_2/*n*-hexane = 2:3, v/v) to afford Br-ACRXTN as a pale-yellow solid (0.12 g, 86%). ^1H NMR (400 MHz, $CDCl_3$): δ 8.59 (d, J = 8.7 Hz, 1H), 8.39 (dd, J = 7.9, 1.8 Hz, 1H), 7.78 (t, J = 7.9 Hz, 1H), 7.56–7.49 (m, 4H), 7.45 (t, J = 7.6 Hz, 1H), 7.31 (dd, J = 8.5, 2.1 Hz, 1H), 7.11 (dd, J = 8.7, 2.3 Hz, 2H), 6.27 (d, J = 9.2 Hz, 2H), 1.66 (s, 6H). ^{13}C NMR (100 MHz, $CDCl_3$): δ 176.43, 151.80, 156.19, 146.65, 139.05, 135.25, 132.75, 129.86, 129.48, 128.29, 126.84, 125.57, 124.46, 121.91, 121.53, 119.40, 118.01, 116.45, 114.35, 36.46, 30.66. MS (MALDI-TOF) (*m/z*): $[M]^+$ calcd. for $C_{28}H_{19}Br_2NO_2$, 560.98; found, 561.05. Anal. (%): calcd. for $C_{28}H_{19}Br_2NO_2$, C 59.92, H 3.41, N 2.50; found: C 59.89, H 3.40, N 2.20. mp: 214 °C.

Synthesis of Br-3-PXZ-XO. To a stirred solution of 3-PXZ-XO (0.0943 g, 0.250 mmol) in THF (5 mL) was added *N*-bromosuccinimide (0.107 g, 0.600 mmol) at 0 °C. The mixture was stirred at that temperature for 1 hour and slowly warmed to room temperature for 8 hours. The mixture was poured into water and then extracted with $CHCl_3$. The combined organic layers were concentrated under reduced pressure. The crude product was purified by silica-gel column chromatography (eluent: $CHCl_3$) to afford Br-3-PXZ-XO as a yellow solid (0.10 g, 75%).^1H NMR (400 MHz, $CDCl_3$): δ 8.58 (d, J = 8.2 Hz, 1H), 8.38 (dd, J = 8.5, 1.6 Hz, 1H), 7.79 (t, J = 7.8 Hz, 1H), 7.55–7.51 (m, 2H) 7.45 (t, J = 7.6 Hz, 1H), 7.33 (dd, J = 8.5, 2.1 Hz, 1H), 6.89 (d, J = 2.3 Hz, 2H), 6.75 (dd, J = 8.7, 2.3 Hz, 2H), 5.90 (d, J = 8.7 Hz, 2H). ^{13}C NMR (100 MHz, $CDCl_3$): δ 176.30, 157.75, 156.14, 144.17, 135.37, 132.24, 130.23, 126.86, 126.42, 125.59, 124.55, 121.95, 121.86, 120.03, 119.08, 118.04, 116.81, 114.64, 113.81. MS (MALDI-TOF) (*m/z*): $[M]^+$ calcd for $C_{25}H_{13}Br_2NO_3$ 534.92; found, 534.65. Anal. (%): calcd. for $C_{25}H_{13}Br_2NO_3$, C 56.11, H 2.45, N 2.62; found, C 56.08, H 2.44, N 2.60. mp: 304 °C.

Supplementary Table 1 Photoluminescence properties of the examined TADF materials.

Materials	τ_{PF} [a] (ns)	τ_{DF} [b] (μs)	Φ_{PL} [c] (%)	Φ_{PF} [d] (%)	Φ_{DF} [e] (%)	$k_{r,S}$ (s^{-1})	k_{ISC} (s^{-1})	$k_{nr,T}$ (s^{-1})	k_{RISC} (s^{-1})
4CzIPN	16	4.6	94	10	84	6.3×10^6	5.6×10^7	1.5×10^4	2.0×10^6
5CzBN	14	3.7	70	8	62	5.7×10^6	6.6×10^7	8.8×10^4	2.3×10^6
4CzBN	1.6	36	62	9	53	5.6×10^7	5.7×10^8	1.7×10^4	1.8×10^5
p-3CzBN	1.2	35	14	10	4	8.3×10^7	7.5×10^8	2.7×10^4	1.2×10^4
2CzPN	25	270	89	51	38	2.0×10^7	2.0×10^7	8.3×10^2	5.6×10^3
Br-4CzIPN	–	–	–	–	–	–	2.5×10^8	–	4.4×10^6
PIC-TRZ2	83	2.7	41	4	37	4.9×10^5	1.2×10^7	2.3×10^5	3.5×10^6
MCz-XT	33	1.2	98	41	57	1.3×10^7	1.8×10^7	2.8×10^4	1.9×10^6
PXZ-DPS	15	2.5	81	22	59	1.5×10^7	5.2×10^7	9.7×10^4	1.4×10^6
DMAC-DPS	20	5.3	86	27	59	1.4×10^7	3.7×10^7	3.6×10^4	5.6×10^5
DBT-BZ-PXZ	39	12	58	16	42	4.1×10^6	2.2×10^7	4.2×10^4	2.6×10^5
DBT-BZ-DMAC	34	20	66	30	36	8.8×10^6	2.1×10^7	2.4×10^4	8.5×10^4
PXZ-DBTO2	27	6.5	49	8	41	2.8×10^6	3.4×10^7	8.5×10^4	9.0×10^5
p-Ac-DABNA	62	1.8	94	64	30	1.0×10^7	5.8×10^6	9.3×10^4	7.2×10^5
ACRFLCN	13	940	67	5	62	3.7×10^6	7.4×10^7	3.7×10^2	1.5×10^4
PXZXO	8	950	42	6	36	7.5×10^6	1.2×10^8	6.5×10^3	6.7×10^4
BTPAc	14	3500	81	33	48	2.3×10^7	4.7×10^7	8.1×10	6.2×10^2
DPE-DPXZ	5.6	543	30	10	20	1.8×10^7	1.6×10^8	1.4×10^3	4.1×10^3
ACRXTN	30	1.6	89	49	40	1.6×10^7	1.7×10^7	1.4×10^5	1.0×10^6
Br-ACRXTN	3.9	1.1	42	25	17	6.4×10^7	1.9×10^8	7.3×10^5	8.7×10^5
3-PXZ-XO	26	0.99	47	43	4	1.6×10^7	2.2×10^7	9.3×10^5	1.7×10^5
Br-3-PXZ-XO	5.0	0.49	45	3	42	6.8×10^6	1.9×10^8	1.2×10^6	2.6×10^7

[a] Lifetime of prompt fluorescence. [b] Lifetime of delayed fluorescence. [c] Total photoluminescence quantum yield. [d] Photoluminescence quantum yield of prompt fluorescence. [e] Photoluminescence quantum yield of delayed fluorescence.

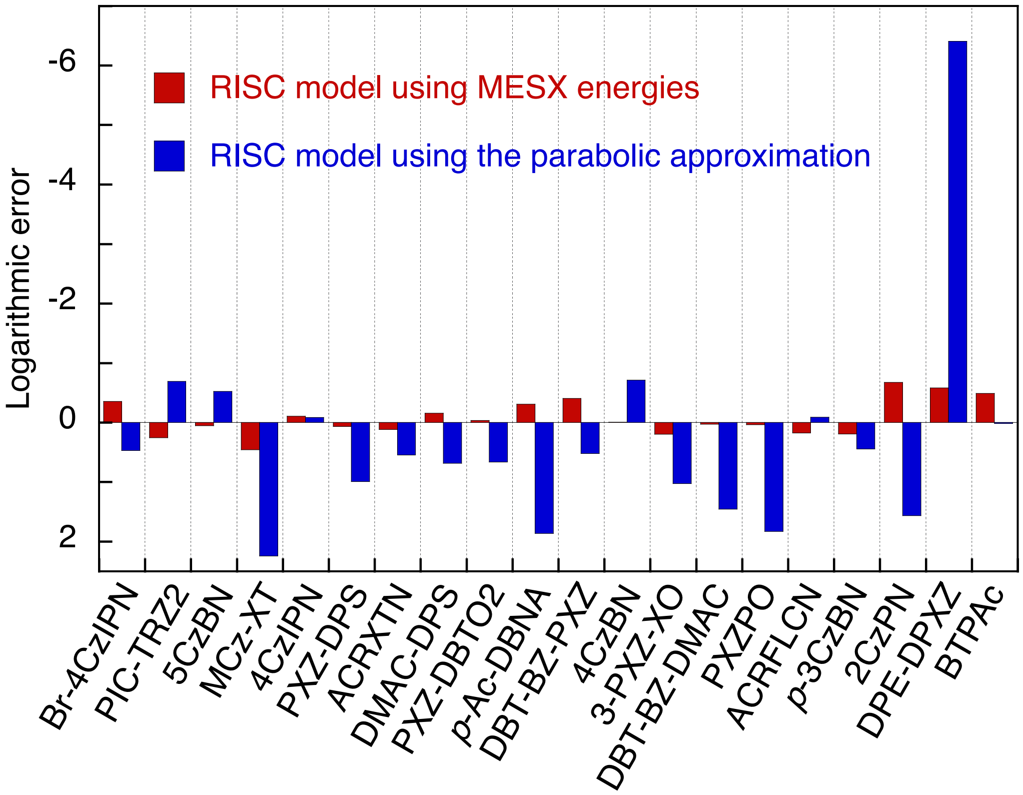

Supplementary Figure 1 Comparison of the logarithmic errors between the experimental and theoretical k_{RISC} values based on the minimum-energy seam of crossing (MESX) and the parabolic approximation.

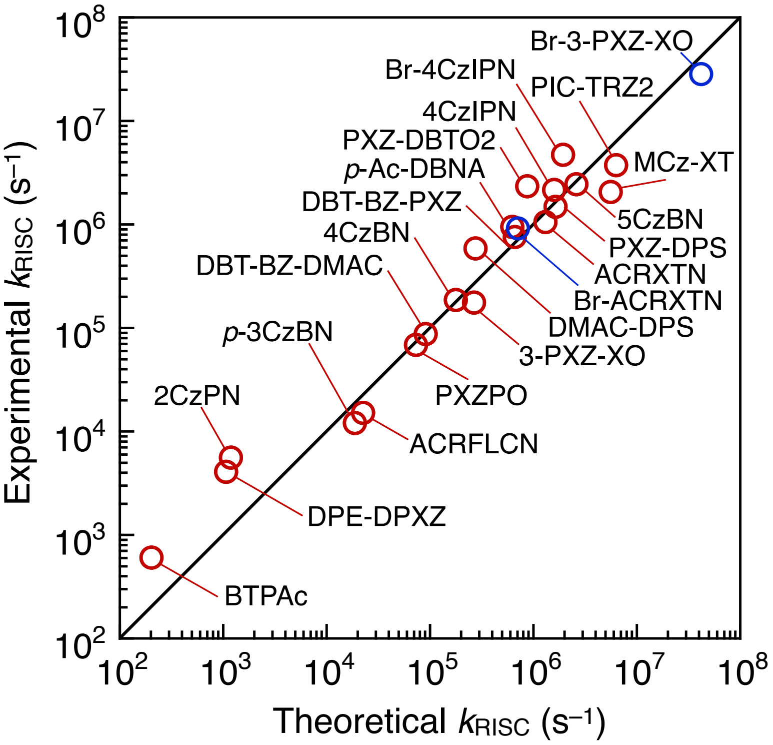

Supplementary Figure 2 Comparison of the experimental and theoretical k_{RISC} values.

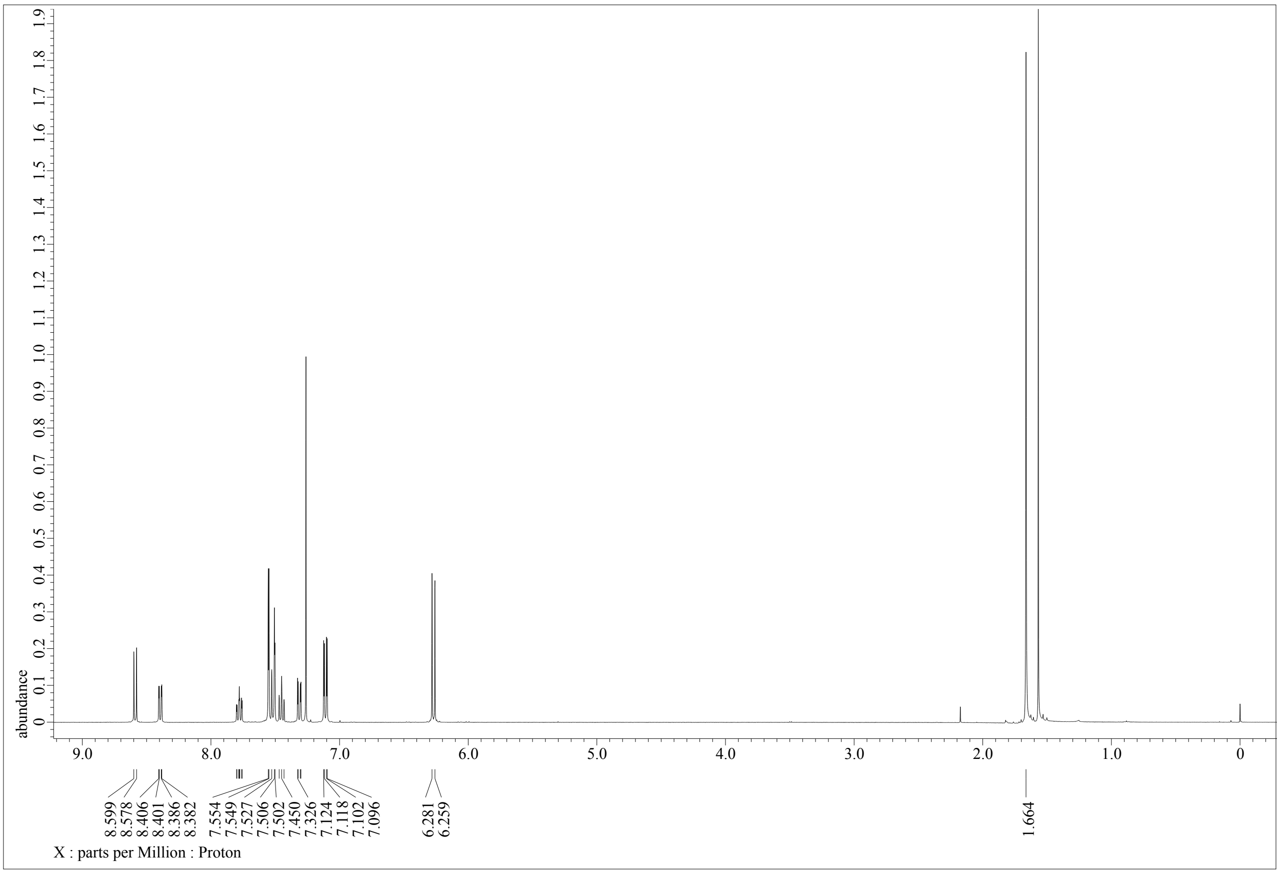

Supplementary Figure 3 ^{1}H NMR spectrum of Br-ACRXTN (400 MHz, $CDCl_3$, 293 K).

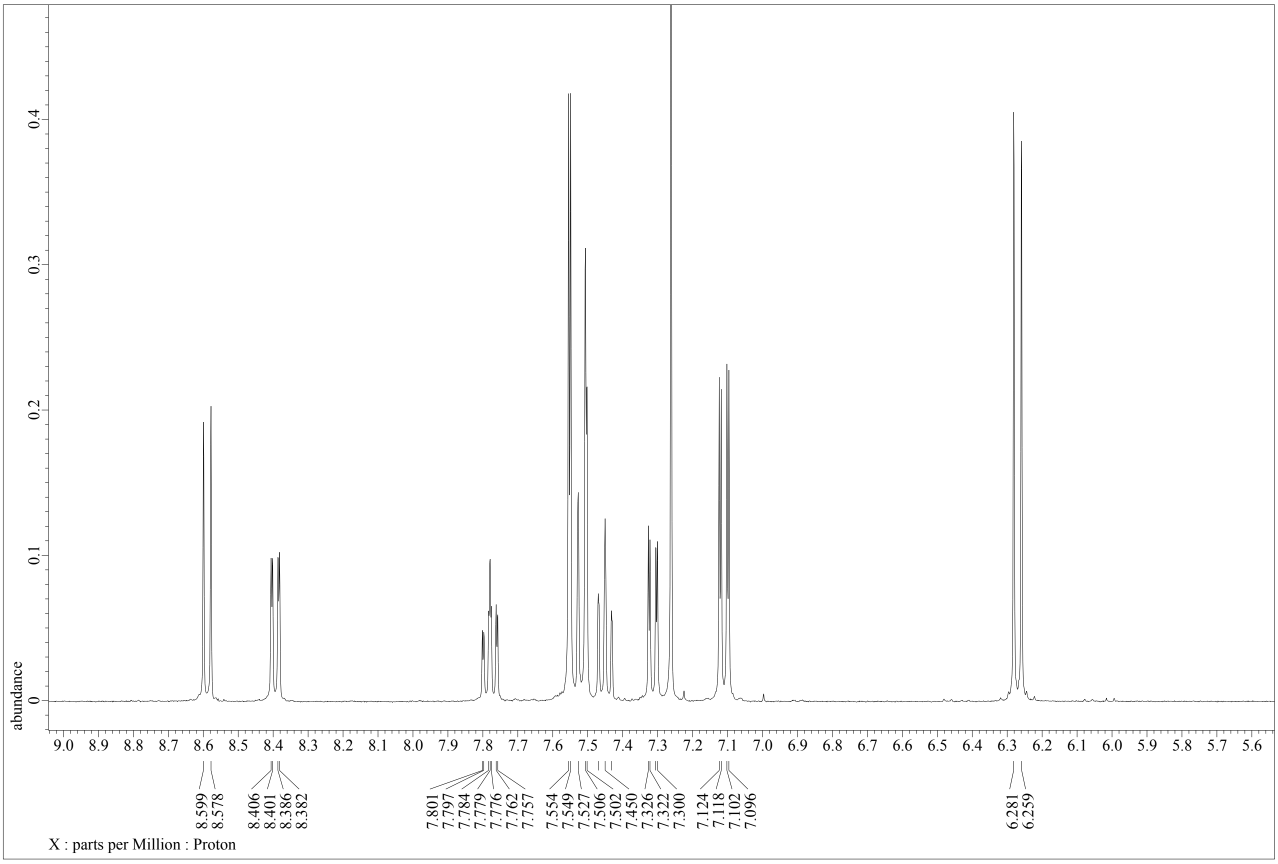

Supplementary Figure 4 Magnified view of the ^{1}H NMR spectrum of Br-ACRXTN (400 MHz, $CDCl_3$, 293 K).

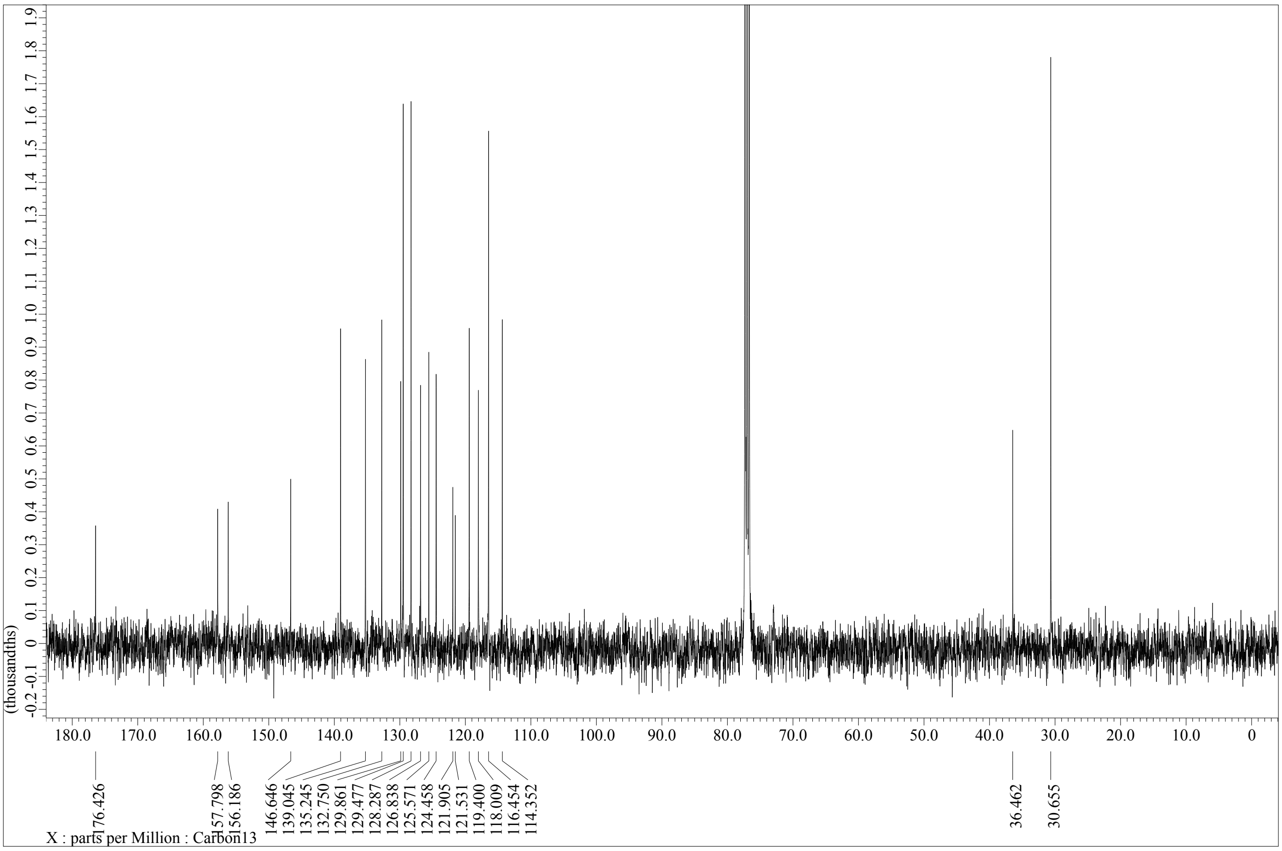

Supplementary Figure 5 ^{13}C NMR spectrum of Br-ACRXTN (100 MHz, $CDCl_3$, 293 K).

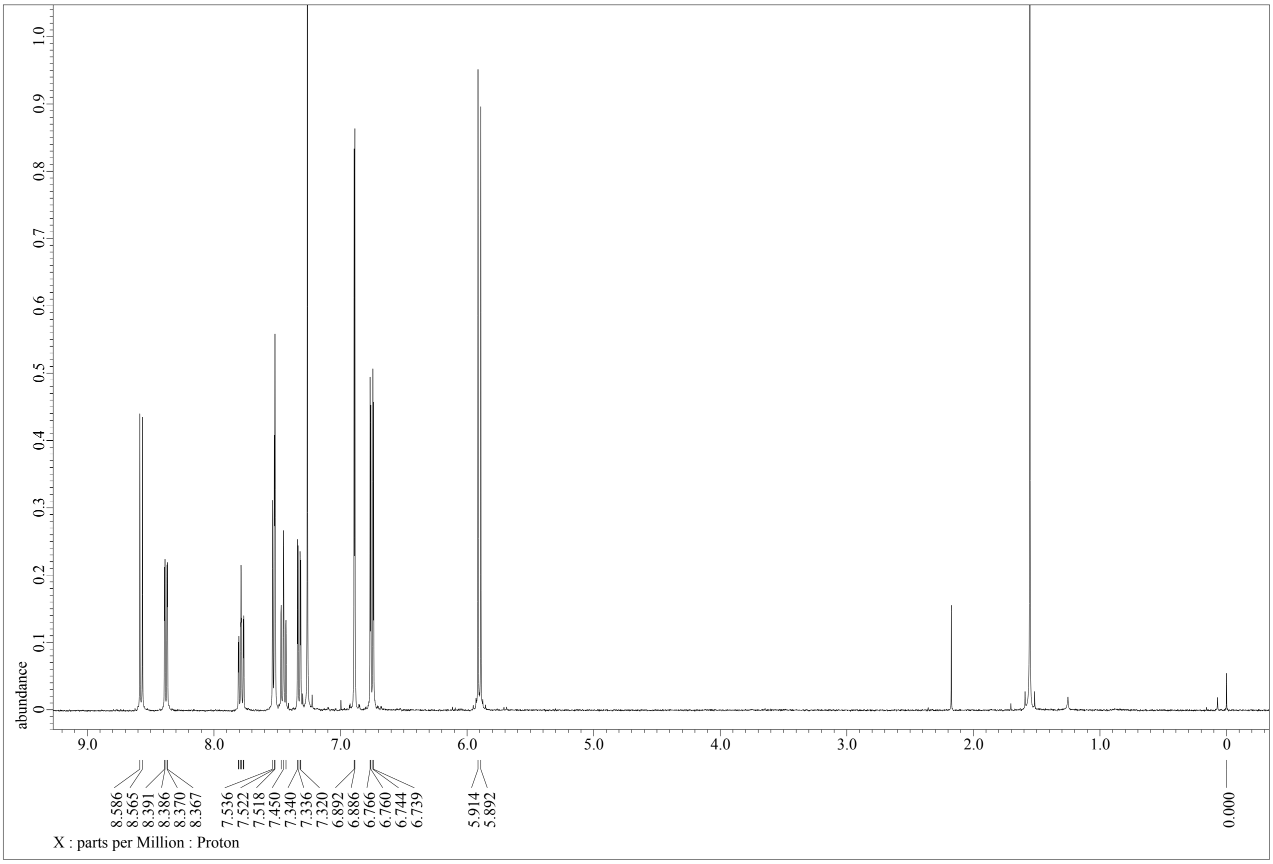

Supplementary Figure 6 ^{1}H NMR spectrum of Br-3-PXZ-XO (400 MHz, $CDCl_3$, 293 K).

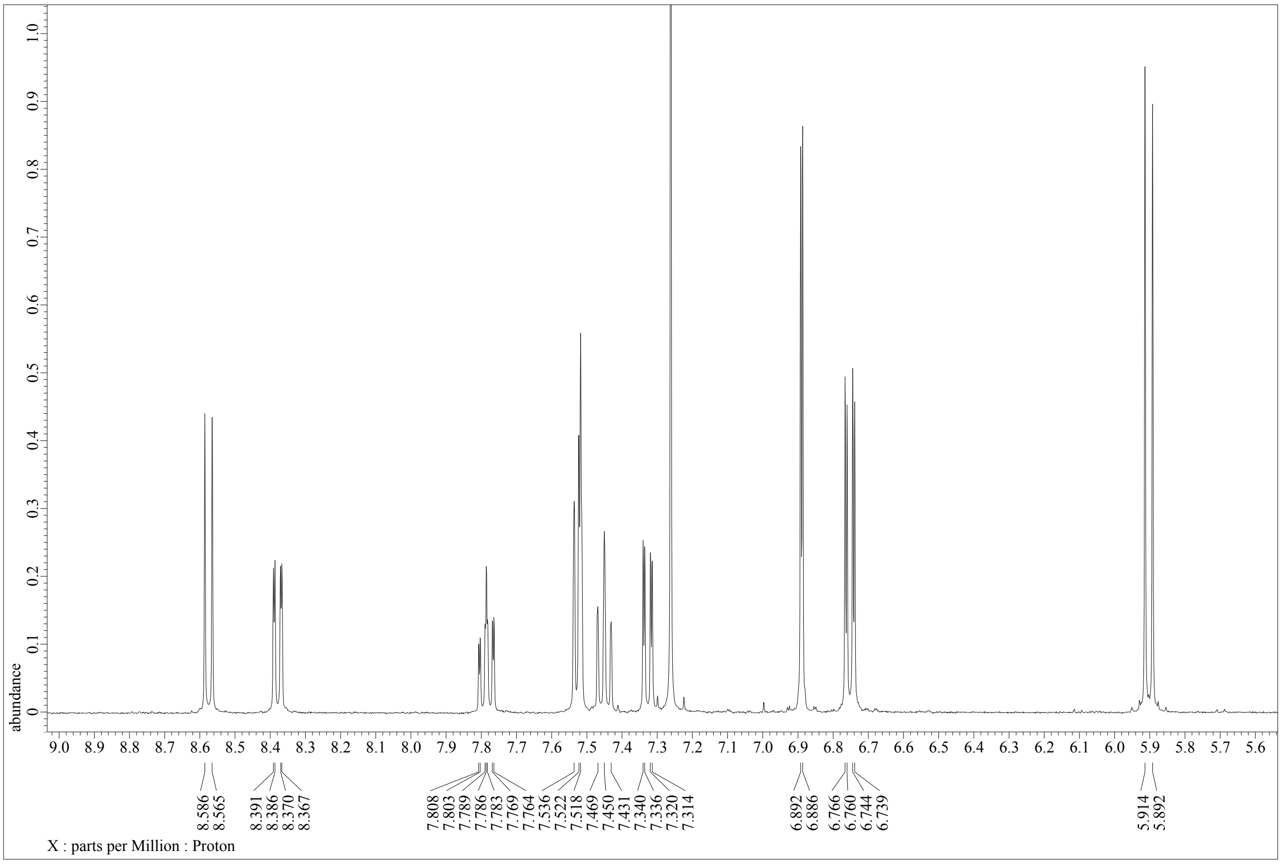

Supplementary Figure 7 Magnified view of the ^{1}H NMR spectrum of Br-3-PXZ-XO (400 MHz, $CDCl_3$, 293 K).

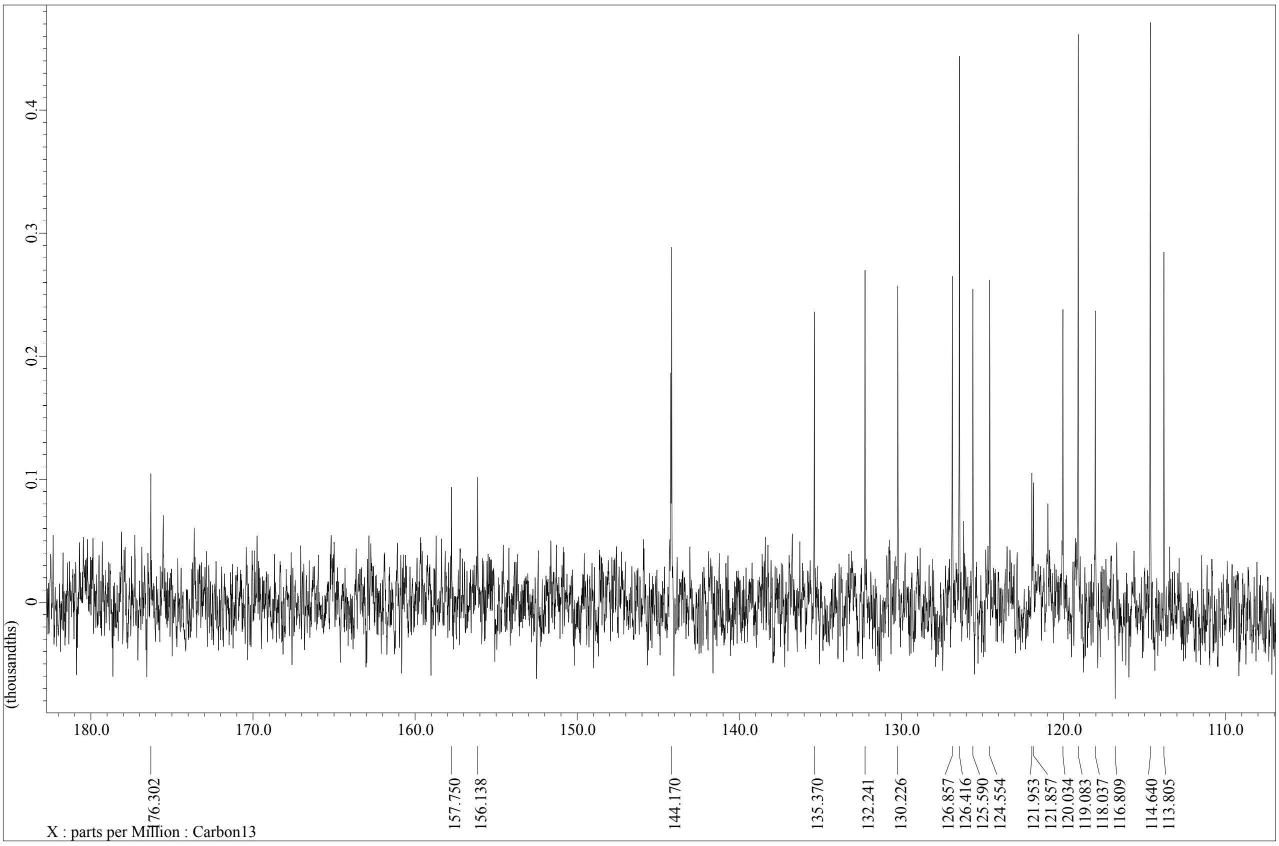

Supplementary Figure 8 ^{13}C NMR spectrum of Br-3-PXZ-XO (100 MHz, $CDCl_3$, 293 K).

2. References

1 Hosokai, T. *et al.* Evidence and mechanism of efficient thermally activated delayed fluorescence promoted by delocalized excited states. *Sci. Adv.* **3**, e1603282 (2017).

2 Zhang, D., Cai, M., Zhang, Y., Zhang, D. & Duan, L. Sterically shielded blue thermally activated delayed fluorescence emitters with improved efficiency and stability. *Mater. Horiz.* **3**, 145–151 (2016).

3 Kim, G. H. *et al.* Controlling the exciton lifetime of blue thermally activated delayed fluorescence emitters using a heteroatom-containing pyridoindole donor moiety. *Mater. Horiz.* **4**, 619–624 (2017).

4 Sato, K. *et al.* Organic luminescent molecule with energetically equivalent singlet and triplet excited states for organic light-emitting diodes. *Phys. Rev. Lett.* **110**, 247401 (2013).

5 Lee, J., Aizawa, N., Numata, M., Adachi, C. & Yasuda, T. Versatile molecular functionalization for inhibiting concentration quenching of thermally activated delayed fluorescence. *Adv. Mater.* **29**, 1604856 (2017).

6 Zhang, Q. *et al.* Efficient blue organic light-emitting diodes employing thermally activated delayed fluorescence. *Nat. Photonics* **8**, 326–332 (2014).

7 Ahn, D. H. *et al.* Effect of various host characteristics on blue thermally activated delayed fluorescent devices. *Org. Electron.* **59**, 39–44 (2018).

8 Guo, J. *et al.* Robust luminescent materials with prominent aggregation-induced emission and thermally activated delayed fluorescence for high-performance organic light-emitting diodes. *Chem. Mater.* **29**, 3623–3631 (2017).

9 Higginbotham, H. F., Yi, C.-L., Monkman, A. P. & Wong, K.-T. Effects of ortho-phenyl substitution on the rISC rate of D–A type TADF molecules. *J. Phys. Chem. C* **122**, 7627–7634 (2018).

10 Meng, G. *et al.* Isomeric bright sky-blue TADF emitters based on bisacridine decorated DBNA: impact of donor locations on luminescent and electroluminescent properties. *Adv. Opt. Mater.* **7**, 1900130 (2019).

11 Méhes, G., Nomura, H., Zhang, Q., Nakagawa, T. & Adachi, C. Enhanced electroluminescence efficiency in a spiro-acridine derivative through thermally activated delayed fluorescence. *Angew. Chem. Int. Ed.* **51**, 11311–11315 (2012).

12 Duan, C. *et al.* Multi-dipolar chromophores featuring phosphine oxide as joint acceptor: a new strategy toward high-efficiency blue thermally activated delayed fluorescence dyes. *Chem. Mater.* **28**, 5667–5679 (2016).

13 Oh, C. S., Lee, H. L., Hong, W. P. & Lee, J. Y. Benzothienopyrimidine as a co-planar type rigid acceptor for high external quantum efficiency in thermally activated delayed fluorescence emitters. *J. Mater. Chem. C* **7**, 7643–7653 (2019).

14 He, Z. *et al.* Sky-blue thermally activated delayed fluorescence material employing a diphenylethyne acceptor for organic light-emitting diodes. *J. Mater. Chem. C* **6**, 36–42 (2018).

15 Yurash, B. *et al.* Photoluminescence Quenching Probes Spin Conversion and Exciton Dynamics in Thermally Activated Delayed Fluorescence Materials. *Adv. Mater.* **31**, 1804490 (2019).

16 Nakanotani, H. *et al.* High-efficiency organic light-emitting diodes with fluorescent emitters. *Nat. Commun.* **5**, 4016 (2014).

17 Zhang, Y. *et al.* Supramolecular structure-dependent thermally-activated delayed fluorescence (TADF) properties of organic polymorphs. *J. Phys. Chem. C* **120**, 19759–19767 (2016).
